# Estimation of cell membrane properties and erythrocyte red-ox balance in patients with metabolic syndrome

**DOI:** 10.1007/s11033-012-2017-x

**Published:** 2012-10-08

**Authors:** Edward Kowalczyk, Jan Kowalski, Jan Błaszczyk, Łukasz Gwoździński, Julita Ciećwierz, Monika Sienkiewicz

**Affiliations:** 1 Department of Pharmacology and Toxicology, Medical University of Lodz, Lodz, Poland; 2 Department of Basis and Pre-Clinical Sciences, Medical University of Lodz, Lodz, Poland; 3 Department of Internal Medicine and Cardiac Rehabilitation, Medical University of Lodz, Lodz, Poland; 4 Department of Medical and Sanitary Microbiology, Medical University of Lodz, pl. Hallera 1, 90-647 Lodz, Poland

**Keywords:** Metabolic syndrome, Erythrocytes, Cell membranes, Red-ox balance

## Abstract

Metabolic syndrome (MS) is associated with occurrence of the many cardiovascular risk factors such as atherogenic dyslipidemia, visceral fat distribution, arterial hypertension and pro-thrombotic and pro-inflammatory status. In our study the effect of disorders that appear in MS on red-ox balance and erythrocyte cell membrane properties were estimated. The study comprised 50 patients with diagnosed MS and in 25 healthy subjects. Content of thiobarbituric acid reactive substances (TBARS) and catalase, superoxide dismutase and glutathione peroxidase activity were estimated in red blood cells. Moreover, conformation status of membrane proteins, membrane fluidity and osmotic fragility were evaluated. MS was found to manifest: (1) the increase of the concentration of TBARS in erythrocytes with no statistically significant differences in antioxidant enzymes activity, (2) disorders in the structure of erythrocyte cytoskeleton proteins, (3) the increase in membrane lipids fluidity at the depth of 5th and 12th carbon atom of fatty acid hydrocarbon chain and significantly decreased fluidity at the depth of 16th carbon atom, (4) increased erythrocyte osmotic fragility.

## Introduction

Numerous clinical and epidemiological observations related to metabolic syndrome (MS) result in more and more frequent molecular as well as pathophysiological and therapeutical investigations that concern especially the nature of the relation between obesity, diabetes mellitus Type 2, arterial hypertension, dyslipidemia and atherosclerotic process and its cardiovascular complications [1]. Endothelial dysfunction is thought to play a crucial role in the progression of atherosclerotic changes.

Oxidative stress related to hypertension, diabetes mellitus and hypercholesterolemia are among factors that cause endothelial damage [2–4]. In an early stage of atherogenesis enhanced adhesive molecules expression, increased adhesion and chemotaxis of monocytes, lymphocytes and platelets are observed [5, 6]. Many published prospective studies stated [7–10], that the risk of cardiovascular complications in atherotrombosis is proportional to the degree of endothelial dysfunction. It seems that red cells in addiction to endothelium can imply atherosclerotic complications. An important feature of an erythrocyte is its viscoelasticity that is its ability of changing shape but its volume remains unchanged [11, 12]. This ability depends among others on red cell shape, membrane fluidity and internal cell viscosity [13–15]. Normal structure of plasmatic membrane of erythrocytes conditions their most important functions: membrane enzymes activity, transport of ions and non-ionic substances, osmotic stability, oxygen diffusion, membrane receptors activity. Decreased erythrocyte viscoelasticity leads to blood flow impairment and worsening of tissue perfusion [13, 16].

The aim of the study was to determine cell membrane properties and erythrocyte red-ox balance in patients with MS.

## Material

The study comprised 50 so far untreated patients (24 women and 26 men), aged 18–75 years (mean 55.9–11.82 years), with MS, hospitalized at the Department of Internal Medicine and Cardiac Rehabilitation, Teaching Hospital No:5 in Lodz. MS was defined according to NCEP/ATPIII [17] criteria and was diagnosed when three or more risk factors were present: central obesity (circumference of waist at the umbilical level: men >102 cm, women >88 cm; serum triglycerides >150 mg/dl or treatment; serum HDL-cholesterol concentration (HDL-C): men <40 mg/dl, women <50 mg/dl or treatment; blood pressure ≥130/85 mm Hg or treatment; fasting glycemia ≥110 mg/dl or treatment).

The control group consisted of 25 healthy subjects (12 women and 13 men) comparable as regards age (mean 54.24 ± 12.84 years).

All the study population gave their informed consent for the inclusion into the study. The study was approved by the Bioethics Committee of Medical University in Lodz, No: RNN/257/05/KB from June 26, 2005.

Characteristics of patients with MS taking a part in the study. The group of patients with MS demonstrated the most frequent occurrence of visceral obesity (92 %) followed by arterial hypertension (86 %), increased triglycerides concentration (72 %), fasting hyperglycemia (48 %), while the most seldom observed component was decreased HDL-C (44 %).

Visceral obesity occurred significantly more frequently than increased triglycerides and glucose levels and decreased HDL-C. Hypertriglycerydemia was observed significantly more often in comparison to the increased glucose and reduced HDL-Cs. Prevalence of elevated blood pressure was significantly more frequent than increased glucose and decreased HDL-Cs.

In the study group, the highest percentage (44 %) were patients with four MS components. Three of the components were found in 40 % of patients, while the presence of all five components was shown in 16 % of patients.

All patients with MS presented high cardiovascular death risk (≥5 %) determined according to SCORE algorithm.

## Methods

### Preparation of erythrocytes

Whole blood was centrifuged and plasma as well leukocyte layer, were removed. The remaining erythrocyte suspension was washed three times with ice-cold PBS solution of pH 7.4 and centrifuged at 3,500 rpm for 10 min (Sigma 3k15). Then, a suspension of 50 % hematocrit value was obtained for studied erythrocytes.

### Isolation of erythrocyte membranes

Erythrocyte membranes were isolated with the modified method of Dodge [18]. Erythrocyte suspension was lysed using 20 mmol/l using phosphate buffer of pH 7.4 and centrifuged. Then, the procedure was repeated using the same solution of the concentration 10 and 5 mmol/l in order to wash off the hemoglobin from the erythrocyte ghosts.

### Physical state of membrane proteins

Conformation changes of membrane proteins were estimated with the electron paramagnetic resonance method using MSL and ISL spin labels. 2 μl of both labels in ethanol solution were added to 1 ml of erythrocyte membrane suspension and the samples were incubated at 4 °C for 60 min. The excess of not bound label was washed out with ice-cold PBS solution of pH 7.4, until EPR signal disappeared.

The ratio *h*_w_/*h*_s_ (*h*_w_—the height of the amplitude of a line from the population of weakly immobilized spin label residue of label to the height of amplitude of strongly immobilized component—*h*_s_) was calculated from the obtained spectra of MSL attached to membrane proteins. In case of ISL the mobility of label bound to proteins was calculated as the relative rotation correlation time τ_c_ [19–21] from the equation:

$$ \tau_{\text{c}} = kw_{0} \left[ {\left( {\frac{{h_{0} }}{{h_{ - 1} }}} \right)^{{ - \frac{1}{2}}} - 1} \right] $$

where: *k*–6.5 × 10^−10^, *w*_0_–width of middle line of EPR spectrum, *h*_0_–height of middle line of EPR spectrum, *h*_−1_–height of high-field line of EPR spectrum.

### Analysis of erythrocyte membrane fluidity

Fluidity of erythrocyte lipid bilayer was estimated by electron paramagnetic resonance method using fatty acids doxyl derivatives (5, 12, 16-doxylstearic) as spin labels. 1 μl of each label in ethanol was added into 1 ml of erythrocyte suspension samples and then incubated for 30 min at room temperature. The ethanol concentration in erythrocyte suspension did not exceed 0.1 %.

Spectra were obtained for all three labels and the ratio of the height of the low-field line amplitude (*h*_+1_) to the height of the middle line amplitude (*h*_0_) was calculated [8, 9]. The measurements were performed at room temperature with Bruker ESP 3000E apparatus [20, 21].

### Determination of erythrocyte osmotic fragility

Erythrocyte osmotic fragility was determined by spectrophotometric method with the use of Beckman DV 650 apparatus. Red blood cells suspension was added to the NaCl solution of decreasing molar concentration: from 150 to 55 mmol/l. Absorbance measurement was performed in a supernatant at the wave length 540 nm. The percentage of hemoglobin was calculated from the equation:

$$ H\% = \frac{{A_{\text{x}} - A_{0} }}{{A_{\text{w}} - A_{0} }}\; \times \;100\;\% $$

where: *A*_x_–sample absorbance, *A*_0_–absorbance for physiological concentration NaCl = 155 mmol/l, *A*_w_–absorbance after total erythrocyte hemolysis in distilled water.

Parameter C_50_, indicating NaCl concentration in which there comes to hemolysis of 50 % of erythrocytes, was calculated from the obtained curves of osmotic fragility.

### Antioxidative enzymes

Erythrocyte MDA concentration was measured according to Placer et al. method [22]. Erythrocyte SOD-1 activity was determined according to the method of Misra and Fridovich [23]. Erythrocyte GSH-Px activity was measured according to the method of Little et al. [24] using coumene hydroperoxide as a substrate. Erythrocyte catalase (CAT) activity was measured according to Beers et al. [25].

## Statistical analysis

The obtained results were subjected to statistical analysis using *t*-Student and Mann–Whitney tests. The testing was performed at the level of significance *p* < 0.05.

## Results

**Fig. 1:**
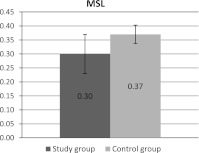
H_w_/h_s_ parameter of maleimide label (*MSL*) bound to control erythrocyte membranes and erythrocytes from patients with metabolic syndrome. Means with standard deviations are presented

Two labels, maleimide (MSL) and iodoacetamide (ISL), covalently bound to protein thiol groups, mainly of the spectrin/actin complex were used to investigate physical status of membrane proteins. The ratio of *h*_w_/*h*_s_ was calculated from EPR spectra of MSL attached to membrane protein. This parameter is a sensitive indicator of protein physical state. In the study group erythrocytes the decrease of this parameter was observed as compared to the controls (statistically significant differences) (Fig. 1).

**Fig. 2:**
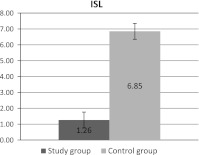
Differences in the rotational correlation time (τ_c_) of iodoacetamide label (*ISL*) bound to erythrocyte membrane proteins between *study* and *control group*. Means with standard deviations are presented

Changes in membrane proteins structure were also observed in the case of iodoacetamide label. Statistically significant differences in the rotational correlation time of the label bound to membrane proteins are shown in Fig. 2.

**Fig. 3:**
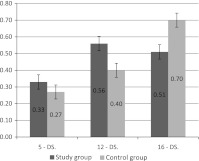
Parameter h_+1_/h_0_ for 5-DS, 12-DS and 16-DS labels incorporated into erythrocyte membrane lipids in *control* and *study group*. Means and standard deviations are presented

Three spin labeled fatty acids 5-DS, 12-DS and 16-DS, having paramagnetic group at different carbon atoms of hydrocarbon chain, were used to estimate erythrocyte lipid membrane fluidity. The ratio of the height of low field amplitude to the height of the spectrum middle line (*h*_+1_/*h*_0_) was calculated from spectra of labeled fatty acids incorporated to lipid membrane. The obtained results (Fig. 3) demonstrated statistically significant increase in the ratio *h*_+1_/*h*_0_ for label 5-DS and 12-DS. In the case of 16-DS label significantly decreased fluidity was observed.

**Fig. 4:**
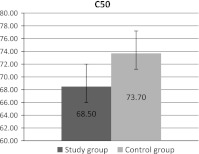
Parameter C_50_ changes in erythrocytes in patients and controls. Means and standard deviations are presented

The erythrocyte osmotic fragility was also investigated in patients and in the control group. Our study demonstrated the decrease of C_50_ parameter in the study group (statistically significant differences). The results are presented in Fig. 4.

In patients with MS statistically significant increase of TBARS content was observed in erythrocytes, while there were no significant changes in antioxidant enzymes activity: CAT, superoxide dismutase (SOD) and glutathione peroxidase (GPX).

Parameters of red-ox balance of erythrocytes in patients and controls are presented in Table 1.

**Table 1:** Red-ox balance of erythrocytes

Group	TBARS (μmol/gHb)	CAT (U/Hb)	SOD (U/gHb)	GSHPx (U/gHb)
Control	0.22 ± 0.05	12.9 ± 2.6	2182 ± .5 481.5	36.4 ± 4.6
MS	0.36 ± 0.08	14.6 ± 4.3	2588.6 ± 521.8	37.2 ± 3.8
Statistical significance	*p* < 0.05	NS	NS	NS

*NS* no significance

## Discussion

Many previous studies have proved that free radical-mediated reactions are one of the main biochemical mechanisms responsible for initiation and progression of early atherosclerotic changes. These reactions lead to lipoprotein oxidative modification and marked changes in cells, that are involved it atherogenic process [26–30]. Red cells are particularly exposed to free radical action, because as molecular oxygen transporters they are a potential source of reactive oxygen species. In physiological conditions about 0.5–3 % hemoglobin is oxidized daily to met-hemoglobin, which is associated with superoxide free radical release. Moreover, erythrocyte cell membranes contain a lot of polyunsaturated fatty acids, which in environment rich in oxygen and oxygen-derived radicals are oxidized to lipid superoxides. Erythrocytes possess multiple enzymatic and non-enzymatic defence mechanisms to prevent peroxidation reactions, however during oxidative stress they become exhausted. Erythrocyte TBARS concentration can be the indicator of peroxidation intensity in these cells because majority of TBARS are peroxidation end-products such as malondialdehyde [30]. Jayakumari et al. observed in patients with stable and unstable angina increased erythrocyte TBARS concentration in comparison to healthy subjects. The concentration of oxidized peroxidation products depended on ischemic heart disease severity and coexisting risk factors. The highest peroxidation products concentration was found in patients with concomitant hiperlipidemia, diabetes mellitus and subsequently in heavy smokers and patients with arterial hypertension. In our study the 60 % increase of erythrocyte TBARS concentration was observed in patients with MS, in comparison with the control group. It may be supposed, that red cell susceptibility to free radical activity is intensified in patients with MS. The results of Simon et al. [31] in vitro studies were the confirmation of this hypothesis. They investigated erythrocytes from severe asymptomatic hypercholestrolemic men and they proved increased reactivity of red cells to AAPH-2,2′-azobis, 2-amidinopropane hydrochloride-azo-complex, that undergoes complete destruction generating at a constant rate water soluble superoxide radicals. The investigators used two parameters that characterize susceptibility of erythrocytes to oxidation: (a) lag-time (LT)–time necessary for the induction of lipid oxidation; (b) T_50_–time needed for the initiation of erythrocyte hemolysis in 50 % of cases. The statistically significant LT and T_50_ shortening was demonstrated in erythrocytes of hypercholesterolemic patients in comparison to the controls. Basing on Simon’s studies [31–33] it was hypothesized, that elevated erythrocyte susceptibility to reactive oxygen species activity in the early stage of oxidative stress resulted mainly from decreased vitamin E content in cells. Similar results were obtained when susceptibility to lipoprotein LDL oxidation was investigated [29]. In our study vitamin E concentration was not evaluated, but we estimated antioxidative enzymes activity: CAT, SOD and GPX. We noted insignificant increase in the activity of these enzymes, however markedly elevated activity was observed in the younger age group (20–30 years) and markedly lower (even as compared to the control group) in the older age group (about 70 years). This observation can confirm the antioxidant system exhaustion in these patients followed by the increase of peroxidation products—TBARS. The majority of investigators postulate, that lipid peroxidation decreases lipid fluidity and stiffens the membrane [34]. In the conditions of increased free radical generation, as it happens in atherosclerosis, MDA and 4-hydroxynoneal concentration increases in plasma and in vessel wall [35]. Lipid peroxidation products can modify physical properties of cell membranes contributing to cell dysfunctions [8]. Introduction of polar superoxide, ketone aldehyde or hydroxide groups into the regions of phospholipid molecules which are localized in the inner side of the lipid bilayer decreases hydrophobicity of the interior of a lipid bilayer membrane and changes bilayer structure. Then there increases in nonspecific the membrane permeability to hydrogen ions and other polar substances for which the lipid bilayer interior is a barrier difficult to pass. Peroxidation decreases electric potential differences on both sides of the membranes (depolarizes them), disturb lipid membrane asymmetry leading to phosphatidylserine exposition on plasmatic membrane outer surface. In normal conditions phosphatidylserine is only kept on the inner-leaflet, the cytosolic side, of cell membranes [36, 37]. Lipid peroxidation causes also inhibition of the activity of membrane enzymes and transporting proteins e.g. (Ca^+2^, Mg^+2^) ATP-ase (calcium pump), Na^+^K^+^(sodium–potassium pump) [37]. In our study apart from changes in red-ox balance, conformation changes in membrane cytoskeleton proteins as well as in membrane lipid fluidity and erythrocytes osmotic fragility were found in patients with MS. Conformation status pf membrane cytoskeleton proteins and membrane lipid fluidity affect erythrocyte shape, size and osmotic fragility. In the study on the conformation of membrane proteins, mainly spectrin/actin complex two spin label (MSL, ISL) were used. EPR spectra of protein bound labels point to the disturbances in the structure of erythrocyte cytoskeleton proteins in patients that can result from oxidative stress and/or changed protein-lipid interactions. These can be conformation changes of protein structure and/or oxidation of thiol groups which are most susceptible to oxidation. Modifications in spectrin/actin complex may affect the membrane cytoskeleton structure. The differences in membrane protein structure observed in the study group can be related to the increase in membrane lipid fluidity at the depth of 5th and 12th carbon atom of fatty acid hydrocarbon chain that shows the modification of polar parts of phospholipids in surface and hydrophobic region of lipid membrane cell monolayer (a half of monolayer). The increase in erythrocyte membrane lipid fluidity was also observed in model studies with *t*-butyl hydroperoxide which initiates peroxidation process [38]. Then, marked decrease in membrane lipid fluidity was observed at the depth of 16th carbon atom spin probe. Most likely disturbances of erythrocyte cell membrane fluidity at different lipid monolayer depth observed in our study resulted from changes in proteins/lipids interactions and/or membrane lipid peroxidation as well as differences in their composition. Changes in the structure of plasmatic membrane components are reflected in erythrocyte osmotic fragility. Impaired erythrocytes osmotic fragility in patients with MS can result from enhanced cell membrane elasticity that is probably related to observed disturbances in membrane cytoskeleton protein structure and increase in lipid bilayer fluidity.

## Conclusions

In patients with MS changes in erythrocytes red-ox balance and osmotic fragility as well as changes of membrane protein conformation status and erythrocyte cell membrane fluidity are observed.
